# #Ihaveembraced: a pilot cross-sectional naturalistic evaluation of the documentary film *Embrace* and its potential associations with body image in adult women

**DOI:** 10.1186/s12905-019-0870-7

**Published:** 2020-02-03

**Authors:** Zali Yager, Ivanka Prichard, Laura M. Hart

**Affiliations:** 1 Institute for Health and Sport, Victoria University, Melbourne, Australia; 2 College of Nursing & Health Sciences, Flinders University, Adelaide, Australia; 3 School of Psychology and Public Health, La Trobe University, Melbourne, Australia; 4 Centre for Mental Health, Melbourne School of Population and Global Health, University of Melbourne, Melbourne, Australia

**Keywords:** Health promotion, Body image, Film, Embrace

## Abstract

**Background:**

The aim of this project was to examine the qualitative responses of adult women who had seen the feature-length documentary film ‘*Embrace*’. In addition, to establish the potential for the documentary to be used as an intervention to improve adult body image, a naturalistic study was conducted to examine whether any differences on measures of body image were apparent among women who had, versus those who had not, seen the film.

**Method:**

Participants were 1429 women aged 18–77 who were members of the Facebook group ‘Body Image Movement’ facilitated by Taryn Brumfitt, who also directed the documentary *Embrace*. Participants completed a cross-sectional online questionnaire regarding whether they had seen the film, their perceptions of the impact of the film on their lives and body image, and a range of standardized scales measuring psychological wellbeing.

**Results:**

Overall, the majority of participants had seen the film (*n* = 1053, 73.7%). Qualitative analysis of open-ended data asking about the changes participants made after viewing the film revealed that a large proportion (44.1%) felt they had higher levels of body appreciation and body confidence, many reported engaging less in dieting (19.6%), and some reported lowered disordered eating (2.8%), since seeing *Embrace*. Women who had seen the film also reported significantly higher levels of body appreciation (Body Appreciation Scale; medium effect size), and significantly lower levels of internalization of body ideals, self-objectification, body shame, and dietary restraint, than women who had not seen the film.

**Conclusions:**

Adult women reported numerous positive responses to their viewing of the film. Future experimental research should explore the efficacy of *Embrace* as a brief and engaging intervention for improving body image in adult women.

## Background

The majority of women are dissatisfied with their bodies [1]. This can result in a wide range of physical and mental health issues, including smoking [2], depression [3], and poorer quality of life [4]. Body appreciation and positive body image are not just the opposite of body dissatisfaction, but refer to a respect, love, and gratitude for the body, and what it can do [5]. Body appreciation is known to increase with age [6], and women with higher levels of body appreciation are more likely to have higher self-esteem, and engage in positive health behaviours such as physical activity and intuitive eating [5].

The extensive impact of negative body image on physical and mental health has led researchers to develop intervention programs to address this public health issue. However, in the past 20 years, the development of interventions for improving body image have focused mostly on adolescents and college-aged women. While some school-based [7] and university [8] interventions have been effective, there are very few programs to improve body image for women who are over the age of 25. In their review of body image interventions among women in mid-life (aged 35–55), Lewis-Smith and colleagues [9] identified only 11 interventions, of which 64% demonstrated a significant improvement on at least one measure of body image at post-test. The most effective interventions (with improvements at post-test and follow-up) for this age group utilized Cognitive-Behavioral Therapy [CBT], and Acceptance and Commitment Therapy [ACT]; while interventions with improvements at post-test only used mindfulness, or physical activity such as dance and yoga [9]. Interventions ranged in length from 5.5 to 155 h, and 70% of the effective interventions were delivered by trained facilitators [9]. For example, *Set Your Body Free,* one of the programs found to be effective over the longer term (significant improvements in body dissatisfaction and disordered eating) in the aforementioned review, was an intensive CBT-based, small-group intervention among women in midlife that ran in 2 hour sessions over 8 weeks [10].

Although effective in creating some behavior change, these interventions are very resource intensive. Sustainability and dissemination of interventions beyond the efficacy phase is generally minimal due to the limited resources of researchers [11]. As such, interventions rarely have significant reach and real-world impact [12]. Furthermore, those most in need of these interventions rarely have ready access to them [13]. To address this, Alan Kazdin (2018) recommends a series of characteristics for innovative interventions to have broad impact including: reach, affordability, convenience, settings, acceptability, flexibility, and task-shifting to a non-professional audience. Films screened in cinemas or online, are a delivery mechanism that meet many of these criteria to engage audiences and create health promotion interventions that have scalable impact.

Many documentary and feature films have been created to expose and explore health-related issues, particularly relating to food and eating (e.g., *That Sugar Film*, *SuperSize Me*, and *Hungry for Change*), and less in relation to mental health and mental illness (e.g., *Thin, Running from Crazy*). Experts and academics often dismiss the impact of feature films about mental illness, and eating disorders especially, as sensationalizing content and exaggeration of the issue [14]. In addition, there are also concerns about the potential iatrogenic effects of films depicting eating and weight issues, for example, obesity films triggering weight stigma, or eating disorder films (e.g., *To the Bone*) triggering dieting or disordered eating behaviors, which is particularly likely when eating disorder films and documentaries explicitly demonstrate harmful behaviors [15]. More recently, research about the Netflix Series *13 Reasons Why*, in which the lead character takes her own life in the final episode, found that search queries related to suicide increased by 19% in the 19 days following the release of the series [16], and hospital admissions for suicide also increased [17]. Film has therefore been relatively controversial and underutilized as an intervention for health promotion specifically for issues relating to eating, weight and body image.

Research investigating the impact of documentary films as mental health promotion interventions is limited. In a recent study, researchers found that state body image was significantly higher at post-test among participants exposed to a 3-min simulated walk through a natural environment (a park), but not among those who experienced a 3-min walk through a built environment [18]. Studies have also used film to reduce stigma against people with a mental illness [19–21], and to reduce weight stigma [22], with promising findings. One study found that a film reduced the extent to which participants blamed individuals with schizophrenia for the disorder, but did not impact general attitudes about the illness or intention to interact with people with schizophrenia [19]. In another study, the 17-min film produced by experts at the Rudd center had a significant improvement on explicit weight bias scores of participants, but no significant change on anti-weight bias [22]. Other reports of using film in teaching and learning at the university level (usually for those in medical, psychology, or psychiatric degrees) also show promise in the use of film to create attitudinal change [23–26]. This evidence indicates that films may be a useful medium for challenging stigma at a surface level, and that feature-length films are not necessarily required to have impact [18].

The documentary *Embrace* was created and directed by Taryn Brumfitt (Founder of the Body Image Movement), via a crowd-funded Kickstarter campaign in 2014. According to the Body Image Movement Website:

“*Embrace is told from the point of view of Taryn as she traverses the globe talking to experts, women in the street and well-known personalities about the alarming rates of body image issues that are seen in people of all body types. In her affable and effervescent style, Taryn bares all (literally) to explore the factors contributing to this problem and seeks to find solutions.”*

*Embrace* uses a message of body acceptance, and perspective-taking, in order to convey the primary message of thinking of bodies as instruments, and not as ornaments. Although not developed with a theoretical framework in mind, the focus on reducing self-objectification does align with other body image interventions. *Embrace* was launched in 2016, and has been distributed in the US, UK, Australia, NZ, and Europe, primarily by individuals hosting screenings at public cinemas, but also on DVD and via online streaming services such as iTunes. *Embrace* is now also freely available to Netflix subscribers in the United States.

The documentary meets many of the criteria set out by Kazdin [13]; it has reach, affordability, convenience, settings, flexibility and task-shifting to a non-professional audience. *Embrace* has reached many individuals all over the world (reach), has been disseminated at scale, and at relatively low cost (affordability). People, and especially women, appear likely to want to consume and access the film more than other evidence-based body image interventions, given film as a medium is interesting and entertaining. The film can be provided in a range of settings – including in an individual’s own home - at flexible times (convenience, flexibility, settings). No treatment providers are required to benefit from watching the film (task-shifting to a non-professional audience), which significantly reduces the cost of dissemination as compared to other psychological interventions requiring specialist facilitation [27]. The only criteria that is not fully met is that of “acceptability to existing providers” [28], as to date there is no evidence regarding women’s opinions of the film.

Thus, the aim of the present study was to explore women’s perceptions of the documentary film *Embrace* and to provide some preliminary data on the body image of women who had and those who had not seen the film*.* We did this by assessing qualitative responses to open-ended questions regarding participants’ descriptions of the impact the film had had on their body image and eating patterns. As the film has content that directly addresses issues surrounding body appreciation, self-objectification, and internalization of the thin ideal, and because these constructs are known to be associated with levels of body shame and dietary restraint [29, 30], we hypothesized that women who had seen the film *Embrace* would have higher levels of body appreciation, lower levels of self-objectification, internalization of the thin-ideal, body shame, and dietary restraint. As the study was cross-sectional, we also sought to determine whether any arising group differences may have been linked to seeing the film, as opposed to women with more positive body image selecting to view the film.

## Methods

The study design involved a cross-sectional survey of adult women using an online community sample, with both open and closed questions, conducted using the survey software Qualtrics. Human Ethics Approval was obtained through the Flinders University Human Ethics Committee, and mirror approval was sought through the Victoria University Human Research Ethics Committee. Recruitment was conducted online through social media networks using a snowball sampling method. The Body Image Movement [BIM] posted the link to the study on their Facebook page (251,264 followers when the survey was conducted in Jan 2018, 254 K followers in June 2019), inviting those who had, and had not seen the film *Embrace* to complete the online questionnaire. Participants were provided with a Participant Information Sheet and indicated their agreement to informed consent statements within the survey software. Followers of BIM were asked to share the public post with their friends to invite them to participate in the research, producing a snowball effect, and recruiting women who were not followers of BIM. Participants were located all over the world but were required to speak English and be 18 years or over to be eligible to participate. Survey completion was open to men and women, but as the film, and the Body Image Movement, largely targets women, we only received one completed survey from a man, and this was removed from the analyses due.

Participants were asked to indicate the date that they saw the film, their ethnicity (‘*Please describe your cultural background’*), age, height, weight, level of education, and the number of children that they had (if any). The online questionnaire consisted of a range of pre-existing, standardized measures that were designed for a larger study, and took approximately 20 min to complete. The order of the standardized scale presentation was consistent across all participants and was not randomized. Other scales asking about role modelling of body image behavior, and postpartum body image (for mothers only), media consumption, and physical activity participation were included in the larger questionnaire (see Additional file 1), but were not analysed for this research. Participants were not remunerated or compensated for their participation in the study, and there was no participant debriefing beyond thanking them for their participation, and the provision of referral details to counselling or helplines to be used if the study had raised any concerns.

### Open-ended questions

Participants were asked to respond to three open-ended questions about their perceptions of the film’s impact: “Tell us your thoughts about the film?”; “What difference (if any) has seeing the film made to your life?” and “What sort of changes (if any) have you made to your life since seeing the film?”

### Positive body image

Positive body image was assessed using the Body Appreciation Scale-2 [BAS-2] [31]. This 10-item measure assesses participant’s acceptance of, positive attitudes toward, and respect for their bodies (e.g., *I feel love for my body*), according to their response on a 5-point scale (never-always), and has been validated and reworded for use with both male and female populations with high internal consistency (α =0.97) [31]. High scores indicate higher levels of body appreciation (scores range from 10 to 50). The BAS-2 indicated high internal consistency within the current sample (α = .956).

### Internalization of the thin ideal

We measured internalization of the thin ideal using the Ideal-Body Stereotyping- Revised [32]. This six-item measure asks participants to indicate the extent to which they agree that certain female body types/shapes are attractive (e.g., *Slender women are more attractive*) on a 5-point scale from strongly disagree to strongly agree. Higher scores reflect higher internalization of the thin ideal. Good internal consistency was observed for this measure in the present study (α = .874).

### Self-objectification

Self-objectification was assessed using the Self-Objectification Questionnaire [30], which asks participants to rank a list of five appearance (e.g., sex appeal, weight) and five competence-based (e.g., energy level, physical fitness) body attributes according to how important each of them are to their physical self-concept. Scores were computed according to the original author’s instructions by summing the ranks for the appearance and competence based attributes respectively, and then creating a difference score, so scores range from − 25 to 25 [30]. Higher scores reflect a greater focus on appearance, and therefore higher levels of self-objectification [30]. Cronbach’s alpha is not possible to calculate as this is a rank-order scale.

### Body shame

Body shame was assessed using the Body Shame subscale from the Objectified Body Consciousness Questionnaire. This 8-item scale (e.g., *When I can’t control my weight, I feel like there must be something wrong with me*) asks a range of questions relating to how participants feel about themselves in relation to their weight and appearance, with responses on a 7-point scale (strongly disagree-strongly agree). High scores on this measure indicate higher levels of body shame, and indicate that participants feel like they are a bad person if they do not conform to societal expectations of their bodies [33]. In the current sample, the measure indicated adequate reliability (α = .685).

### Dietary restraint

Dieting behavior was assessed using the 10-item Dietary Restraint subscale of the Dutch Eating Behavior Questionnaire [34]. This widely used scale asks participants to respond to items such as “*when you have eaten too much, do you eat less than usual the following days*” on a 5-point scale (never-very often). High scores indicate higher levels of dietary restraint. This scale has acceptable internal consistency (α = .95) and test–retest reliability (*r* = .82) and has been found to correlate negatively with observed caloric intake in the natural environment [32, 34]. The measure indicated high internal consistency in the current study sample (α = .928).

Data analysis procedures for the open-ended data utilized established protocols [35]. Two researchers familiarized themselves with the data. They independently generated codes for the first 50 responses to each question. They compared, discussed, and refined the codes until they reached agreement on the coding framework. A further two different independent researchers then coded responses to all questions. Kappas were calculated for inter-rater agreement and the coding was revised until acceptable agreement (> 0.7) was reached across all data, by highlighting discrepancies and discussing these until agreement was reached between the two coders. Content analysis was conducted across codes to generate response frequencies.

All quantitative data was analyzed in SPSS version 24. Frequencies, means and standard deviations were obtained in order to characterize the sample. Average scores for key body image measures were calculated as per scoring standard instructions. A Multiple Analysis of of Covariance [MANCOVA] was performed to assess differences in body image measures between participants who had seen *Embrace* and participants who had not, controlling for age, BMI, and education level. Prior to the analysis, a number of tests were conducted to ensure the data met assumptions for the MANCOVA procedure. P-P plots indicated residuals were approximately normal and no significant covariances were observed between groups (Box’s M = 242.68, *p* > .05). Significant moderate collinearity was observed between body image variables (*r* = −.317 to *r* = .716, *p* < .001) and no multivariate outliers were observed. Due to the exploratory nature of the study, the alpha level for significance was divided by 3 (*p* = .017) to adjust for multiple comparisons for the three primary variables investigated for effects on body image (age, motherhood, and having viewed the film *Embrace*). Age, BMI, and economic status are known to be associated with differences in body image attitudes among adult women [2, 6], so we controlled for these variables (using level of education as a proxy for economic status) in our analyses. Effect sizes (ES) were obtained using calculations for eta squared *(η*^*2*^*)* and where appropriate, Hedge’s *g.*

## Results

Overall, 1429 women completed the questionnaire. Although drawn from over 15 countries around the world, most respondents identified as Caucasian (*n* = 1317, 91.5%) and a small number identified as European or Asian (*n* = 50, 3.5%; *n* = 23, 1.6%). Over half of respondents had children (*n* = 858, 60.0%). Age ranged from 18 to 77. On average participants were aged 41 years (*SD* = 10.69; Median age = 40). The majority of participants had seen the film (*n* = 1053, 73.7%), and followed the Body Image Movement on Facebook (*n* = 1177, 82.4%). Among those who had seen the film, the majority (45.5%) had seen it within the last 12 months, 36.3% has seen it within the last 6 months, 10.8% had seen it within a month, and 2.6% had seen it within the last week. Almost all of the participants (94.5%) had only seen the film once, and 4.8% had seen it more than once.

### Opinions about *Embrace* and perceived impact

In total, 1099 women provided answers to the question on overall thoughts about the film, and 1075 women responded to either question on whether viewing the film had made a difference to their lives or whether they had made any changes since viewing the film. For all three items, each potential thematic code was recorded as Yes/No in terms of being present, for each of the open-ended responses from women, and as such, more than one code may have been present in each woman’s response.

Data relating to general thoughts about the film are presented in Table 1. Overall, 97.4% of women expressed positive thoughts about the film. The most common code that emerged in the thoughts about the film was that they enjoyed it (present in 46.4% of responses). The second (40.8%) was that women had an emotional response to the film, reporting they felt “moved”, “inspired”, “empowered” and “motivated” to make change as a result of seeing *Embrace*.

**Table 1:** Frequencies of codes in response to women’s ‘thoughts about the film’

Theme	Code	Proportion %(n)	Sample response
Neutral	No strong opinion either way	1.5% (16)	*“I had mixed emotions. First, I felt very empowered and very proud of my body and what it can do...but I also feel like I’m doing something wrong when I have negative feelings about my body, which is part of life. I’m not perfect and it is hard to be body positive all the time especially when your body doesn’t do what you want it to.”*
Negative	Expresses some concerns about content	1.1% (12)	“*Sharing the fact that models eat cotton balls and all have eating disorders is sensationalizing*” “*It was confronting as a person who has an eating disorder*”
Enjoy	Loved the film	46.4% (510)	*“I thought it was a good movie”* “*I thought it was totally 100% awesome*”
Awareness	Disturbing to know that women feel this way	5.2% (57)	*“Was very confronting but really struck a chord with me.”* *“It was sad to see and hear how many women view themselves and wonderful to see those embracing who they are.”*
	Importance of the message being out there to increase awareness of the issue and start conversations	28.8% (317)	*“Important message about how we women are told, constantly, insistently, and insidiously, how our bodies are never good enough, ….”* *“Very positive, changing, inspirational, well done so that even though there [are] sad moments it’s funny and happy too. After my friends and I saw it we wanted to keep talking about it and also I encouraged a lot of other female friends to see it”*
Personal Importance	Feeling of not being alone	9% (99)	“*It was enlightening to know most women all feel the same about their bodies*.” “*It was like I’m not the only one to feel like this.”*
	Assisted body acceptance/body appreciation	7.9% (87)	*“I love it and it inspired me to change my life and view of my body. That momentum is still going months later.”*
	Emotional response - Felt moved inspired, empowered and motivated to make change	40.8% (448)	“*Impactful, inspirational, emotional”* *“Very empowering”* *“Powerful and relieving. I felt that I was ok to be me for once in my life.”* *“It was so moving to me as a woman who has fought with my own body image throughout my life, and has watched so many women close to me struggle. Now that I’m the mom of a pre-teen daughter, this message is especially timely. I’m so glad I saw it with her at just this moment in her life, when she was beginning to hate her body-- after the show, she told me that the film CHANGED her LIFE, that it gave her a whole new perspective on body image. ... The tickets to the screening might have been the best money I ever spent.”*

In response to questions about any differences that seeing the film had made on participants’ lives, and what changes may have been made as a result of seeing the film, women frequently mentioned changes in their own knowledge, attitudes or behaviors (personal change; see Table 2) and changes resulting in their behavior towards others (such as towards their peers or children; see Table 3).

**Table 2:** Frequencies of Personal Change codes

Theme	Code	Proportion %(n)	Sample response
Neutral	No impact	11.4 (123)	*“No significant impact to my own life.”* *“Unfortunately not much”* *“Immediately after I felt good about myself. Now however 6 months later I am back to having awful self-esteem and a negative body image.”*
Changes in Knowledge	Feeling that I am not alone- struggles are valid	6.7% (72)	*“Made me realize that I am not the only one that feels the way I do about my body,”* *“That most women have insecurities.”*
	Change in knowledge or awareness	11.4% (123)	*“Not only have I started looking at myself differently... but I pushed this out for women in my community where how fair and how thin you are is valued more than your personality.”*
	Enhanced critique of societal standards	8.8% (95)	*“I think more about what I see in the media in terms of it being very one dimensional in the type of women/people portrayed.“*
Changes in Attitudes	Valuing qualities other than appearance	7.5% (81)	*“Feeling less concerned with superficial thoughts of my own body image and appreciating more of how clever my body is...”* *“I’m going to make a conscious effort to consentrate [*sic*] on the important things and not my weight.”*
	Self-compassion and being less judgmental or engaging in less social comparison	36.9% (400)	*“I have really tried to let some of that stuff go and just live life without worrying how other people see me.”* *“Stopping my inner critic when I find I’m being negative towards myself and my appearance. Remembering some of the people in the film and their rise above adversity.”*
	Body acceptance and body confidence	44.1% (478)	*“It has made me think differently about my body”* *“It put a louder voice/ memory/ pathway in my system to value self-acceptance more than a # on the scale”*
Changes in Behavior	Behavioral indications of reduced body dissatisfaction	16.2% (176)	*“A big difference! Lets just say i just bought a swimsuit and im determined to wear it without shorts and a top over it! I want to set an example that porcelain skin, cellulite and stretch marks are normal and nothing to be ashamed of!!”* *“Have been trying to ‘embrace’”*
	Reduced dieting, engaging in self-care for health	19.6% (212)	*“I have embraced the non-diet culture.”* *“I’m not trying to restrict food or exercise to get thin.”* *“Less guilt associated with food.”*
	Reduced ED Symptomology	2.8% [30]	*“I am clean for the first time for 12 years from bulimia, that’s something New for me*

**Table 3:** Frequencies of Change Towards Others codes

Theme	Code	Proportion %(n)	Sample response
Changing thoughts about others	Reduced judgement of, and greater acceptance if diversity in others	9.8% (106)	*“I am less likely to judge others.* *“And I’m being a lot less judgmental of others and their bodies.”* *“I’ve also really looked hard at how I unconsciously judge others too.”*
Change social environment	Being conscious of not contributing to this issue	4.9% (53)	*“I feel like I am more sensitive to others feelings. I don’t commiserate when my friend’s complain about their body’s. I tell them they’re beautiful.”* *“I don’t let my friends talk badly about themselves,”*
Spreading the message and empowering others	Impact on Parenting	13.9% (151)	*“I recommend it to fellow Moms and am trying to figure out a way to have it screened at my daughters school.* *“It has caused me to rethink how I see myself and what words I put out there that my daughter and other women hear”*
	Impact on Professional Capacity	4.5% (49)	*“has influenced my practice also as a dietitian working with families”* *“huge, eye opening as a health care worker already committed to the healthy -at- every - size paradigm. Inspired to keep fighting and being optimistic about body esteem for our younger people.”*
	Becoming an ambassador for this issue	10.2% (110)	*“It made it more real and urgent for me the need to speak up and tell the world: “hey, it’s about time we change these things “.”* *“It makes me want to double down on my work to help women feel better about themselves.”*

Participants reported a wide range of reactions to the film. In terms of their personal changes, most commonly participants reported feeling like they had greater body appreciation (44.1%), greater self-compassion (36.9%), less dieting behavior (19.6%), and less disordered eating symptomatology (2.8%) as a result of seeing the film. In terms of engagement with others, participants reported feeling like the film positively impacted on their parenting (13.9%), being less judgmental of others (9.8%), and on their decision to become a body image ambassador (10.2%; a voluntary role associated with the Body Image Movement that involves sharing the messages of *Embrace*).

### Differences between women who had viewed *Embrace* and those who had not on body image outcomes

Results indicated significant differences on body image scores between women who had seen *Embrace* and women who had not (*F*(5,1177) = 20.71, Wilk’s λ = .915, *p* < .001; see Table 1). When controlling for age, Body Mass Index [BMI], and education level, women who had seen the film had greater body appreciation (with the largest effect size, accounting for 7.2% of the difference in scores) than women who had not watched the *Embrace* film (*F*(1,1190) = 82.04, *p* < .001*, η*^*2*^.072) (Table 4).

**Table 4:** Mean scores on body image measures adjusted for age, BMI and education

	Film seen M(SD)	Film not seen M(SD)	MANCOVA	Effect Size *η*^*2*^*/d*	Effect Size
Body appreciation	3.45 (.03)	2.94 (.05)	*F* = 82.04, *p* < .001	.072/0.56	Medium
Body shame	3.79(.05)	4.21 (.09)	*F* = 18.61, *p* < .001	.017/0.26	Small
Self-objectification	−12.24 (.43)	−7.79(.75)	*F* = 26.48, *p* < .001	.024/0.31	Small
Internalization	3.03 (.03)	3.29 (.05)	*F* = 19.01, *p* < .001	.018/0.27	Small
Dietary restraint	2.67 (.03)	2.92 (.05)	*F* = 16.46, *p* < .001	.014/0.24	Small

## Discussion

This study aimed to examine women’s perceptions of the film *Embrace* as well as whether the self-reported body appreciation, body shame, and self-objectification of women who had and had not seen the film. The qualitative data indicated that *Embrace* had a strong emotional impact on many women, and as a result of viewing the film, many reported making many changes in their lives, both in terms of their personal knowledge attitudes and behaviors, and in terms of their engagement with others. Some women wrote responses of up to 300 words in length, describing in depth, the confronting, emotional experience of viewing the film, and what this meant for them personally, and the people that they work or live with. The personal actions reported in response to the film were as diverse as: participants being less judgmental of themselves, joining body positive groups on social media, buying and wearing swimsuits without being self-conscious, deciding not to have cosmetic surgery, and focusing on nutrition instead of dieting. Many women detailed how seeing the film has helped them in parenting their daughters (and to a lesser extent, their sons), by being more aware of how they speak about their own, and other peoples’ bodies in front of others, and being motivated to be a positive body image role model. Positive parental role modelling of body attitudes is especially important as research indicates that it is critical in enhancing children’s body image [36, 37]. Interestingly, people from a variety of backgrounds (i.e., bus drivers, teachers, and psychologists), reported applying what they learned from the film in their professional capacity demonstrating the potential reach of the film.

In relation to differences between women who had and had not seen the film, we found that women who had seen the film *Embrace*, reported higher positive body image, lower self-objectification, body shame, internalization of the thin ideal, and dietary restraint, than women who had not seen the film. Whilst the present study was cross-sectional in nature, these findings are promising and point to the need for future research to examine the impact of the film Embrace using a controlled experimental design. The strength and consistency of the effect sizes (*d*’s 0.24–0.56) are impressive. These effect sizes for body image measures (*d*’s 0.26–0.56) are comparable to, but overall still smaller than, the effect sizes reported in the review of more intensive interventions for women in midlife (*d*’s 0.23–1.72), where the average *d* of the effective studies was 0.79 [9]. The effect size for dietary restraint (d = 0.24) found in this research was lower than the effect size given in an evaluation of *Set Your Body Free* using the same measure (d = 0.90) that was delivered over a 6-month period [9, 10]. These results indicate that *Embrace* may be useful as a body image intervention for adult women, though future experimental research is required to establish a causal effect and to assess how scores on relevant body image and eating disorder measures change over time before and after seeing the film.

Like all research, this study has limitations. Although very few studies of adult women have such a large community sample with such rich qualitative data, the cross-sectional nature of the research, and the convenience and snowballing sampling method used means that we cannot be sure that the differences in scores observed between those who had, and those who had not seen the film, are attributable to viewing the film. It is possible that there was selection bias across groups whereby women who had higher body image to begin with may have chosen to watch the film. However, it is also possible, given the strength of the qualitative responses and perceived changes reported by women, that those with poor body image sought out the film to feel better about themselves. It is also possible that, recruiting women for the study with the purpose of gathering feedback about the film, and asking them questions about the film, may have resulted in socially desirable responding, however the trait measures of body image and eating behavior should not have been affected by this as the scoring of those measures would not be obvious to participants. Future research should utilize experimental designs to compare scores of women before and after they see the film in comparison to an active control film, to accurately measure the direct impact of *Embrace* and establish whether it fully meets the criteria for efficacy [38]. A further limitation of this research is that we were not able to conduct sensitivity analyses on responses according to participant country, race, or ethnicity. However, this would be warranted in future research, to understand whether *Embrace* is effective for adult women and for which women in particular.

Despite the limitations, the current findings, and in particular, the qualitative responses, indicate that there is a high degree of consumer acceptability of the film. Once efficacy can be established through more rigorous experimental research design, this will contribute to acceptability by professionals, and thus ensure that the *Embrace* Documentary film meets all of the criteria for innovative interventions that can have a broad impact [13].

## Conclusion

This research evaluated adult women’s responses to the feature-length documentary film *Embrace*. There are relatively few interventions for adult women available to promote healthy body attitudes, and our results provide preliminary evidence to suggest that women who view *Embrace* report more positive body image and personal change as a result of the film. Future research is needed to further examine the impact of this film over-time and hence its value as a population-level, sustainable and affordable intervention for improving women’s body attitudes.

## Supplementary information

**Additional file 1:.** Embrace Survey containing all questions and measures completed via Qualtrics online.

## Data Availability

The datasets used and/or analysed during the current study are available from the corresponding author on reasonable request.

## Abbreviations

ACT: Acceptance and Commitment Therapy
BAS-2: Body Appreciation Scale-2
BIM: Body Image Movement
BMI: Body Mass Index
CBT: Cognitive-Behavioral Therapy [CBT]
MANCOVA: Multiple Analysis of Variance
SD: Standard Deviation
