# Supplementary material for: #Ihaveembraced: a pilot cross-sectional naturalistic evaluation of the documentary film Embrace and its potential associations with body image in adult women
Source: BMC Womens Health. 2020 Feb 3;20:18. doi: 10.1186/s12905-019-0870-7 (PMC6998832; doi:10.1186/s12905-019-0870-7)
Supplement: Supplementary file 1 — Additional file 1:. Embrace Survey containing all questions and measures completed via Qualtrics online. [file 12905_2019_870_MOESM1_ESM.pdf]

Information Sheet

Welcome!
Thank you for your interest in this study.
Please read the following information sheet and then click next to participate.

INFORMATION SHEET
'Exploring the impact of the Body Image Movement and 'Embrace' the film'

Researchers:
Dr Ivanka Prichard
College of Nursing & Health Sciences
Flinders University
Ph: +61 8 8201 3713

Dr Zali Yager
College of Education
Victoria University
Ph: +61 3 9919 4555

Description of the study:
This study is part of the project entitled 'Exploring the impact of the Body Image Movement and 'Embrace' the film. This project will investigate opinions of the film, 'Embrace', and its association with body image. This project is supported by the College of Nursing & Health Sciences at Flinders University and the College of Education at Victoria University.

Purpose of the study:
This project aims to evaluate the impact of 'Embrace' the documentary and the Body Image Movement on the body image and psychological wellbeing of adolescent and adult women. It will do this by comparing the experiences of women who have and have not seen 'Embrace'.

What will I be asked to do?
You will be invited to complete an online questionnaire that assesses your opinions about the film 'Embrace' as well as measures of body image, eating behaviour, and thoughts about parenting (if applicable). The questionnaire will take about 20-30 minutes to complete. Participation is entirely voluntary.

What benefit will I gain from being involved in this study?
The sharing of your experiences will allow future body image researchers to understand ways to more effectively promote positive body image to large groups of people. We are very keen to deliver resources which are as useful as possible to people.

Will I be identifiable by being involved in this study?
We do not need your name and you will be anonymous. Be assured that any information provided will be treated in the strictest confidence and none of the participants will be individually identifiable in any resulting report or other publications. You are, of course, entirely free to discontinue your participation at any time or to decline to answer particular questions.

Are there any risks or discomforts if I am involved?
The film has been screening in Australia since September 2016 and has received a very positive response from girls and women 12 years and older. The researchers anticipate few risks from your involvement in this study; however given the nature of the project some participants could experience emotional discomfort involved with answering questions related to body image. If any emotional discomfort is experienced please contact Lifeline on 13 11 14 or the Butterfly Foundation on 1800 33 4673 for support that may be accessed free of charge by all participants. If you have any concerns regarding anticipated or actual risks or discomforts, please raise them with the researchers.

How do I agree to participate?
Participation is voluntary. You may answer 'no comment' or refuse to answer any questions and you are free to withdraw from the study at any time without effect or consequences. Completion of the online questionnaire will be considered as informed consent. If you are under the age of 18, please contact the researchers for information on how to participate and to gain parental consent by emailing ivanka.prichard@flinders.edu.au or zali.yager@vu.edu.au.

How will I receive feedback?
On completion of the project, a summary of the findings will be available on bodypositivemums.org.

Thank you for taking the time to read this information sheet and we hope that you will accept our invitation to be involved.

This research project has been approved by the Flinders University Social and Behavioural Research Ethics Committee (Project number 7481). For more information regarding ethical approval of the project the Executive Officer of the Committee can be contacted by telephone on 8201 3116, by fax on 8201 2035 or by email human.researchethics@flinders.edu.au

Demographic Information

How old are you?

Please specify your cultural background?

- Caucasian
- Asian
- Aboriginal or Torres Strait Islander
- African
- Other (please specify)

What is your postcode/zipcode?

What is your highest level of education? (i.e. Year 10, Year 12, Bachelor, Masters, Doctoral)

- Year 10
- Year 11
- Year 12
- Bachelor
- Masters
- Doctoral
- Other

Engagement with the Body Image Movement

Do you follow the Body Image Movement on:

	Yes	No
Facebook	○	○
Twitter	○	○
Instagram	○	○

To what extent have you engaged with the Body Image Movement on Social Media (Facebook, Twitter, Instagram?)

Not Engaged										Highly Engaged
0	1	2	3	4	5	6	7	8	9	10
○	○	○	○	○	○	○	○	○	○	○

Have you seen the film Embrace

- ○ Yes
- ○ No

We are interested in your thoughts about the film 'Embrace' and the Body Image Movement in general.

When did you see the film:

Please spend a moment and tell us what your thoughts are about the film:

What difference (if any) has seeing the film made to your life?

What sort of changes (if any) have you made to your life since seeing the film?

If you have children, how has seeing the film changed (if at all) the way you speak about your body in front of your child(ren)?

What are your thoughts about the film being shown in secondary schools to young people aged 12 - 17 years?

Any other thoughts?

Media consumption

On a typical day how often do you check Facebook (even if you are logged on all day)?

- not at all
- once a day
- every few hours
- every hour
- every 30 minutes
- every 10 minutes

- every 2 minutes

Overall, how long do you spend on Facebook on a typical day?

- 5 minutes or less
- 15 minutes
- 30 minutes
- 1 hour
- 2 hours
- 4 hours
- 6 hours
- 8 hours
- 10 hours or more
- Not at all

Overall, how long do you spend on Twitter on a typical day?

- 5 minutes or less
- 15 minutes
- 30 minutes
- 1 hour
- 2 hours
- 4 hours
- 6 hours
- 8 hours
- 10 hours or more
- Not at all

Overall, how long do you spend on other social media outlets (e.g. Instagram, Snapchat) on a typical day?

- 5 minutes or less
- 15 minutes
- 30 minutes
- 1 hour
- 2 hours
- 4 hours
- 6 hours
- 8 hours
- 10 hours or more
- None

Overall, how long do you spend on the internet on a typical day (not including social media)?

- 5 minutes or less
- 15 minutes
- 30 minutes
- 1 hour
- 2 hours
- 4 hours
- 6 hours
- 8 hours
- 10 hours or more
- None

In a typical week, how much time do you spend watching TV (including online streaming such as Netflix and DVDs/movies) each day (**NOT** for work or homework)?

- 5 minutes or less
- 15 minutes
- 30 minutes
- 1 hour
- 2 hours
- 4 hours
- 6 hours
- 8 hours
- 10 hours or more
- None

How long do you spend reading magazines during a 7-day week?

- 5 minutes or less
- 15 minutes
- 30 minutes
- 1 hour
- 2 hours
- 4 hours
- 6 hours
- 8 hours
- 10 hours or more
- none

Body Image

The following questions relate to feelings about your body. Please indicate how much each statement applies to you by selecting the appropriate number:

	Never 1	Seldom 2	Sometimes 3	Often 4	Always 5
I respect my body	◯	◯	◯	◯	◯
I feel good about my body	◯	◯	◯	◯	◯
I feel that my body has at least some good qualities	◯	◯	◯	◯	◯
I take a positive attitude towards my body	◯	◯	◯	◯	◯
I am attentive to my body's needs	◯	◯	◯	◯	◯
I feel love for my body	◯	◯	◯	◯	◯
I appreciate the different and unique characteristics of my body	◯	◯	◯	◯	◯
My behavior reveals my positive attitude toward my body; for example, I hold my head high and smile	◯	◯	◯	◯	◯
I am comfortable in my body	◯	◯	◯	◯	◯
I feel like I am beautiful even if I am different from media images of attractive people (e.g. models, actresses/actors).	◯	◯	◯	◯	◯

For parents

Please indicate your gender

- ◯ Male
- ◯ Female

Do you have children?

- ◯ Yes
- ◯ No

How many children do you have?

____

What is the gender and age of each of your children (if any)?

	Gender		Age		Biological child?
	Male	Female	Years	months	Select if Yes
Child 1	◯	◯			◯
Child 2	◯	◯			◯
Child 3	◯	◯			◯

	Gender		Age		Biological child?
	Male	Female	Years	months	Select if Yes
Child 4					
Child 5					
Child 6					

Please enter any additional explanatory information about your children here:

For each of the following scenarios please rank the items according to how likely you would be to react in the way described, towards your child.

Note, that we do not expect you to have experienced each scenario, but ask that you think about what you might do if it occurred.

Please rank from MOST likely (1) to LEAST likely (4) BY DRAGGING THE ITEMS UP AND DOWN THE LIST. If you want each response in the place it is displayed, you need to move some options around for the software to recognize that you have answered the question.

1. In the supermarket, your child points to an overweight person and asks "why are they so big?"

You explain to your child that everyone is different and that is OK.

You comment on the size of the person and explain to your child that it's not healthy.

You ignore the comment because you're not really sure how best to tackle it.

You think this is a good opportunity to tell your child about the importance of keeping trim and watching what they eat to avoid getting big.

2. You see your child look at themself in the mirror and say they hate their nose.

You tell your child that it doesn't matter because they can change it when they are older.

You don't worry about it because they are too young to have serious concerns about their appearance.

You don't know what to say, so say nothing.

You ask your child why they have said that and listen to what they have to say.

3. Your child is bigger than average. They come home from playing at their cousin's house and say that they were called "fat".

You suggest to your child that given the cousin's big size they shouldn't be making such comments.

You start limiting how much your child is eating to help them lose weight.

You reassure your child that body size doesn't matter, as long as they are healthy.

You tell your child to just forget about it because you're not sure how to respond.

4. An old friend of yours drops in, bringing along their child who is the same age as yours. You notice they don't play well together. After they leave, you ask your child why. Your child says because they thought the other child's freckles made them look funny.

You tell your child off for being mean.

You agree with your child that freckles do look funny, but they should still try to play nicely with other children.

You explain to your child that they should try to play together again because you know they will find the visiting child is very kind.

You aren't quite sure what to say, so tell your child to be nicer next time.

5. You are at a party where there are lots of lollies, cakes, and sweets but no fresh food available. You would like to make sure your child doesn't eat too many of these foods.

You explain to your child that the party foods are bad for them and they might gain weight if they eat too many of them.

You allow your child to eat what they want now, but make sure they don't have any party foods for a while to make up for what they eat now.

You allow your child to choose a small number of foods to enjoy, explaining that we need to leave room for dinner later on.

You follow what other parents are letting their children do.

6. You are concerned that your child is overweight.

You try to give more nutritious foods and encourage the whole family to have time each evening where they are physically active together, without telling your child you are concerned about their weight.

You discuss how eating too many of the wrong foods will cause them to become overweight and they should try to eat less.

You aren't sure what to do, so wait to see what happens.

You encourage them not to eat as many snacks and make sure they don't help themselves to second servings at mealtimes.

Please indicate how much you agree or disagree with the following statements:

	Strongly Disagree 1	Disagree 2	Neither Agree nor Disagree 3	Agree 4	Strongly Agree 5

	Strongly Disagree 1	Disagree 2	Neither Agree nor Disagree 3	Agree 4	Strongly Agree 5
I am an excellent role model of positive body image for my child/ren.	◯	◯	◯	◯	◯
I make positive comments about my body in front of my child/ren.	◯	◯	◯	◯	◯
I avoid talking about my body in a negative way in front of my child/ren.	◯	◯	◯	◯	◯
I avoid talking about other people's bodies in a negative way in front of my child/ren.	◯	◯	◯	◯	◯
My child/ren see(s) me engaging in positive, joyful movement.	◯	◯	◯	◯	◯
My child/ren see(s) me enjoying a wide variety of foods without restraint.	◯	◯	◯	◯	◯
I avoid talking about diet and exercise for weight control in front of my child/ren.	◯	◯	◯	◯	◯

Please respond to the following questions:

	Yes	No	Prefer not to answer
Have you been diagnosed with perinatal or postpartum depression?	◯	◯	◯
Are you currently taking medication for perinatal or postpartum depression?	◯	◯	◯

Please indicate how true these items are for you **since having your child(ren)**:

	Since having my child(ren) ...				
	Not at all true for me 1	Untrue for me 2	Neutral 3	True for me 4	Very true for me 5
I have felt more satisfied with my body size and shape	◯	◯	◯	◯	◯
I have attempted to make dietary changes to lose weight	◯	◯	◯	◯	◯
I have felt worse about my looks than I did before children	◯	◯	◯	◯	◯
I have felt satisfied with my weight	◯	◯	◯	◯	◯
I have compared my physical appearance to the appearance of celebrity mums who have their 'pre-baby body back'	◯	◯	◯	◯	◯
I have felt that I look better than the average mother	◯	◯	◯	◯	◯
I have compared my physical appearance to the appearance of other mums that I know or see who have their 'pre-baby body back'	◯	◯	◯	◯	◯
I have initiated new exercise regimes specifically to lose weight	◯	◯	◯	◯	◯
I have considered plastic surgery to alter my appearance	◯	◯	◯	◯	◯
I have felt more physically attractive	◯	◯	◯	◯	◯

Internalisation

What is your height? (If you know it - leave blank if you don’t)

What is your current weight? (If you know it - leave blank if you don't)

What is your ideal weight? (If you know it - leave blank if you do not have one)

Please respond to the following:

	Strongly disagree 1	2	3	4	Strongly agree 5
Slender women are more attractive.	○	○	○	○	○
Women who are in shape are more attractive.	○	○	○	○	○
Tall women are more attractive.	○	○	○	○	○
Women with toned (lean) bodies are more attractive.	○	○	○	○	○
Shapely women are more attractive.	○	○	○	○	○
Women with long legs are more attractive.	○	○	○	○	○

Dietary Restraint

The following questions ask you about your eating behavior. Please indicate how frequently each question is true for you by selecting the appropriate number.

	Never 1	Seldom 2	Sometimes 3	Often 4	Very often 5
If you have put on weight, do you eat less than you usually do?	○	○	○	○	○
Do you try to eat less at mealtimes than you would like to eat?	○	○	○	○	○
How often do you refuse food or drink offered because you are concerned about your weight?	○	○	○	○	○
Do you watch exactly what you eat?	○	○	○	○	○
Do you deliberately eat foods that are slimming?	○	○	○	○	○
When you have eaten too much, do you eat less than usual the following days?	○	○	○	○	○
Do you deliberately eat less in order not to become heavier?	○	○	○	○	○
How often do you try not to eat between meals because you are watching your weight?	○	○	○	○	○

	Never<br>1	Seldom<br>2	Sometimes<br>3	Often<br>4	Very often<br>5
How often in the evening do you try not to eat because you are watching your weight?					
Do you take your weight into account when you eat?					

Objectification

We are interested in how people think about their bodies. The questions below identify 10 different attributes. We would like you to rank order these body attributes from that which has the greatest impact on your physical self-concept, to that which has the least impact on your physical self-concept.

NOTE: It does not matter how you describe yourself in terms of each attribute. For example, fitness level can have a great impact on your physical self-concept regardless of whether you consider yourself to be physically fit, not physically fit, or any level in between.

Please first read over all of the attributes. Then, drag and drop the attribute that is most important to you with 1 being most important and 10 being least important.

WHEN CONSIDERING YOUR PHYSICAL SELF-CONCEPT, HOW IMPORTANT IS...

- Physical coordination?
- Health?
- Weight?
- Strength?
- Sex appeal?
- Physical attractiveness?
- Energy level (e.g. stamina)?
- Firm/sculpted muscles?
- Physical fitness level?
- Measurements (e.g. chest, waist, hips)?

Now indicate how strongly you agree or disagree with each of the following statements in relation to your own life by selec appropriate number from 1, *strongly agree, to 7, strongly disagree. If the statement does not apply to you, you may selec applicable to yourself).*

	Strongly Disagree<br>1	Disagree<br>2	Moderately Disagree<br>3	Neither Disagree nor Agree<br>4	Moderately Agree<br>5	Agree<br>6	Strongly Agree<br>7	A
When I can't control my weight, I feel like there must be something wrong with me.								
I feel ashamed of myself when I haven't made the effort to look as good as I could.								
I feel like I must be a bad person when I don't look as good as I could.								

	Strongly Disagree 1	Disagree 2	Moderately Disagree 3	Neither Disagree nor Agree 4	Moderately Agree 5	Agree 6	Strongly Agree 7	A
I would be ashamed for people to know what I really weigh.								
I never worry that something is wrong with me when I am not exercising as much as I should.								
When I'm not exercising enough I question whether I am a good enough person.								
Even when I can't control my weight, I think I'm an ok person.								
When I'm not the size I think I should be, I feel ashamed.								

Physical activity and sport participation

The last set of questions relate to your current physical activity.
Please complete if applicable.

Q1. Please list a **physical activity/sport** that you currently participate in (if any).

Q1. How **often** do you partake in this activity/sport a week?

Q1. How **long** do you spend doing this activity/sport (on each occasion)?
Please specify in **minutes**.

Q1. **Where** do you perform this physical activity/sport (e.g. at home, beach, etc.)?

Are there any other types of physical activity/sport you engage in currently?

- Yes
- No

Q2. Please list another **physical activity/sport** that you currently participate in (if any).

Q2. How **long** do you spend doing this activity/sport (on each occasion)?
Please specify in minutes.

Q2. How **often** do you partake in this activity/sport a week?

Q2. **Where** do you perform this physical activity/sport (e.g. at home, beach, etc.)?

Are there any other types of physical activity/sport you engage in currently (not mentioned above)?

- Yes
- No

Q3. Please list another **physical activity/sport** that you currently participate in (if any).

Q3. How **long** do you spend doing this activity/sport (on each occasion)?
Please specify in minutes.

Q3. How **often** do you partake in this activity/sport a week?

Q3. **Where** do you perform this physical activity/sport (e.g. at home, beach, etc.)?

Are there any other types of physical activity/sport you engage in currently (not mentioned above)?

- Yes
- No

Q4. Please list a **physical activity/sport** that you currently participate in (if any).

Q4. How **long** do you spend doing this activity/sport (on each occasion)?
Please specify in minutes.

Q4. How **often** do you partake in this activity/sport a week?

Q4. **Where** do you perform this physical activity/sport (e.g. at home, beach, etc.)?

Block 14

Thank you very much for your time! We really appreciate it.

On completion of the project, a summary of the findings will be provided on bodypositivemums.org.
If you have any questions or would like more information, please contact ivanka.prichard@flinders.edu.au or zali.yager@vu.edu.au.

If you experienced any emotional discomfort from answering any questions please contact Lifeline on 13 11 14 or the Butterfly Foundation on 1800 33 4673 for support that may be accessed free of charge.
